# Supplementary material for: An incoherent feedforward loop facilitates adaptive tuning of gene expression
Source: eLife. 2018 Apr 5;7:e32323. doi: 10.7554/eLife.32323 (PMC5903863; doi:10.7554/eLife.32323)
Supplement: Supplementary file 2. — (A) Determination of GAT1 variants binding specificities by Protein Binding Microarray. Each purified protein was assayed on two different microarrays containing different probe sequences (ME or HK). E-scores for the top 8-mer bound by the protein on each microarray were calculated. A PWM was generated for each protein variant based on an alignment of the top ten 8-mers. (B) GAT1 protein fragment sequences used in PBM and EMSA assays. The DNA binding domain is in blue. Flanking sequences included in the expressed protein fragment are in green. (C) Oligonucleotides used to generate duplex DNA for EMSAs. Bold and underlined sequences are GATA sequences bound by GAT1. The minimum match score was set as 0.8 using ‘matchPWM()’ function in ‘Biostrings’ library in R. Negative motifs differ from target motifs only at the underline GATA by replacement with random sequence. [file elife-32323-supp2.docx]

**Supplementary file 2A. Determination of GAT1 variants binding specificities by Protein Binding Microarray.**

| **GAT1 allele** | **Microarray** | **Top 8mer** | **E-score** | **PWM** |
| --- | --- | --- | --- | --- |
| GAT1-wt | ME | TATCGATA | 0.48924 | 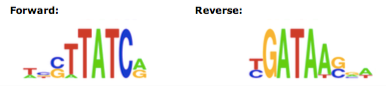 |
| GAT1-wt | HK | TGATATCA | 0.48725 |  |
| GAT1-1 | ME | TAAATTTA | 0.42353 | 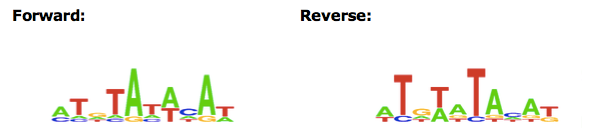 |
| GAT1-1 | HK | ATCTAGAT | 0.42561 |  |
| GAT1-3 | ME | ATGTACAT | 0.44819 | 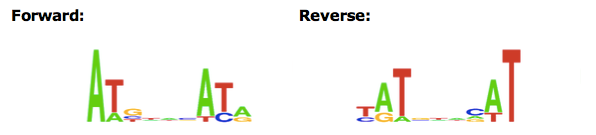 |
| GAT1-3 | HK | ATCTAGAT | 0.46281 |  |

**Supplementary file 2B. GAT1 protein fragment sequences used in PBM and EMSA assays.**

| > **GAT1 (WT)**  MHVFFPLLFRPSPVLFIACAYIYIDIYIHCTRCTVVNITMSTNRVPNLDPDLNLNKEIWD  LYSSAQKILPDSNRILNLSWRLHNRTSFHRINRIMQHSNSIMDFSASPFASGVNAAGPGN  NDLDDTDTDNQQFFLSDMNLNGSSVFENVFDDDDDDDDVETHSIVHSDLLNDMDSASQRA  SHNASGFPNFLDTSCSSSFDDHFIFTNNLPFLNNNSINNNHSHNSSHNNNSPSIANNTNA  NTNTNTSASTNTNSPLLRRNPSPSIVKPGSRRNSSVRKKKPALKKIKSSTSVQSSATPPS  NTSSNPDIKCSNCTTSTTPL**W**RKDPKGLPLCNACGLFLKLHGVT**R**PLSLKTDIIKKRQRS  STKINNNITPPPSSSLNPGAAGKKKNYTASVAASKRKNSLNIVAPLKSQDIPIPKIASPS  IPQYLRSNTRHHLSSSVPIEAETFSSFRPDMNMTMNMNLHNASTSSFNNEAFWKPLDSAI  DHHSGDTNPNSNMNTTPNGNLSLDWLNLNL*  > **GAT1 (*gat1-1; W321L*)**  MHVFFPLLFRPSPVLFIACAYIYIDIYIHCTRCTVVNITMSTNRVPNLDPDLNLNKEIWD  LYSSAQKILPDSNRILNLSWRLHNRTSFHRINRIMQHSNSIMDFSASPFASGVNAAGPGN  NDLDDTDTDNQQFFLSDMNLNGSSVFENVFDDDDDDDDVETHSIVHSDLLNDMDSASQRA  SHNASGFPNFLDTSCSSSFDDHFIFTNNLPFLNNNSINNNHSHNSSHNNNSPSIANNTNA  NTNTNTSASTNTNSPLLRRNPSPSIVKPGSRRNSSVRKKKPALKKIKSSTSVQSSATPPS  NTSSNPDIKCSNCTTSTTPL**L**RKDPKGLPLCNACGLFLKLHGVTRPLSLKTDIIKKRQRS  STKINNNITPPPSSSLNPGAAGKKKNYTASVAASKRKNSLNIVAPLKSQDIPIPKIASPS  IPQYLRSNTRHHLSSSVPIEAETFSSFRPDMNMTMNMNLHNASTSSFNNEAFWKPLDSAI  DHHSGDTNPNSNMNTTPNGNLSLDWLNLNL*  **> GAT1 (*gat1-3, R345G*)**  MHVFFPLLFRPSPVLFIACAYIYIDIYIHCTRCTVVNITMSTNRVPNLDPDLNLNKEIWD  LYSSAQKILPDSNRILNLSWRLHNRTSFHRINRIMQHSNSIMDFSASPFASGVNAAGPGN  NDLDDTDTDNQQFFLSDMNLNGSSVFENVFDDDDDDDDVETHSIVHSDLLNDMDSASQRA  SHNASGFPNFLDTSCSSSFDDHFIFTNNLPFLNNNSINNNHSHNSSHNNNSPSIANNTNA  NTNTNTSASTNTNSPLLRRNPSPSIVKPGSRRNSSVRKKKPALKKIKSSTSVQSSATPPS  NTSSNPDIKCSNCTTSTTPLWRKDPKGLPLCNACGLFLKLHGVT**G**PLSLKTDIIKKRQRS  STKINNNITPPPSSSLNPGAAGKKKNYTASVAASKRKNSLNIVAPLKSQDIPIPKIASPS  IPQYLRSNTRHHLSSSVPIEAETFSSFRPDMNMTMNMNLHNASTSSFNNEAFWKPLDSAI  DHHSGDTNPNSNMNTTPNGNLSLDWLNLNL* |
| --- |

**Supplementary file 2C. Oligonucleotides used to generate duplex DNA for EMSAs**

| **Oligo name** | **Sequence** | **Note** |
| --- | --- | --- |
| MEP2_motif1_F | 5’-CTTTAA**GATAA**GAAA**GATAAGATAA**GACAT-3’ | Target motif1 of MEP2 |
| MEP2_motif1_R | 5’-ATGTCTTATCTTATCTTTCTTATCTTAAAG-3’ |  |
| MEP2_motif1_neg_F | 5’-CTTTAAACGCCGAAAACGCCACGCCGACAT-3’ | Negative motif1 of MEP2 |
| MEP2_motif1_neg_R | 5’-ATGTCGGCGTGGCGTTTTCGGCGTTTAAAG-3’ |  |
| MEP2_motif2_F | 5’-AGTCAAAGCCA**GATAAGATAA**GAAATGTAA-3’ | Target motif2 of MEP2 |
| MEP2_motif2_R | 5’-TTACATTTCTTATCTTATCTGGCTTTGACT-3’ |  |
| MEP2_motif2_neg_F | 5’-AGTCAAAGCCAACGCCACGCCGAAATGTAA-3’ | Negative motif2 of MEP2 |
| MEP2_motif2_neg_R | 5’-TTACATTTCGGCGTGGCGTTGGCTTTGACT-3’ |  |
| DAL80_motif1_F | 5’-CCCGTTTGTCGTT**GATAA**CGAGTTTCCACC-3’ | Target motif1 of DAL80 |
| DAL80_motif1_R | 5’-GGTGGAAACTCGTTATCAACGACAAACGGG-3’ |  |
| DAL80_motif1_neg_F | 5’-CCCGTTTGTCGTTACGCCCGAGTTTCCACC-3’ | Negative motif1 of DAL80 |
| DAL80_motif1_neg_R | 5’-GGTGGAAACTCGGGCGTAACGACAAACGGG-3’ |  |
